# Novel Treatment of Sunstroke

**Published:** 1868-12-15

**Authors:** 


### *Novel Treatment of Sunstroke.*

Dr. F. G. Herron, one of the City Physicians of Cincintati, Ohio (*Med. and Surg. Reporter*), has tried in two cases, with success, the following treatment in sunstroke: Warm water was applied to the head, on cloths, as warm as the skin could bear without injury. Consciousness was very soon restored. Liquor ammoniæ acetatis was administered internally as a stimulant.—*Exchange.*

